# Using self-drilling screws in volar plate osteosynthesis for distal radius fractures: a feasibility study

**DOI:** 10.1186/s12891-016-0972-4

**Published:** 2016-03-10

**Authors:** Alexaner Synek, Lars Borgmann, Hannes Traxler, Wolfgang Huf, Ekkehard Euler, Yan Chevalier, Sebastian F. Baumbach

**Affiliations:** Institute of Lightweight Design and Structural Biomechanics, Vienna University of Technology, Vienna, Austria; Center for Higher Education, TU Dortmund University, Dortmund, Germany; Center of Anatomy and Cell Biology, Department of Systematic Anatomy, Medical University of Vienna, Vienna, Austria; Center of Medical Physics and Biomedical Engineering, Medical University of Vienna, Vienna, Austria; Department of Trauma Surgery, Campus Innenstadt, Ludwig-Maximilians-University, Nussbaumstrasse 20, 80336 Munich, Germany; Department of Orthopedic Surgery, Laboratory for Biomechanics and Experimental Orthopedics, Campus Grosshadern, Ludwig-Maximilians-University, Munich, Germany

**Keywords:** Distal radius, Morphometry, Distal radius fracture, Plate osteosynthesis, Biomechanics

## Abstract

**Background:**

Symptomatic extensor tendon irritation is a frequent complication in volar plate osteosynthesis of distal radius fractures. It is typically caused by dorsal screw protrusion and overdrilling of the dorsal cortex. The use of self-drilling locking screws (SDLS) could overcome both causes. The practical applicability of SDLS depends on two prerequisites: (1) the feasibility of preoperative distal screw length determination, and (2) sufficient primary biomechanical stability of SDLS compared to standard locking screws (SLS).

**Methods:**

We first assessed the feasibility of preoperative screw length determination (1): Distal radius width, depth and distal screw lengths were measured in 38 human radii. Correlations between distal radius width and depth were assessed, a cluster analysis (Ward’s method and squared Euclidean distance) for distal radius width conducted, and intra-cluster screw lengths analyzed (ANOVA). The biomechanical performance of SDLS (2) was assessed by comparison to SLS in a distal radius fracture model (AO-23 A3). 75 % distal screw length was chosen for both groups to simulate a worst-case scenario. Uniaxial compression tests were conducted to measure stiffness, elastic limit, maximum force and residual tilt. Statistics comprised of independent sample t-tests and a Bonferroni correction (*p* < 0.0125).

**Results:**

(1) Distal radius width and depth showed a high correlation (*R*^*2*^ = 0.79; *p* < 0.001). Three distal radius width clusters could be identified: small <34 mm; medium 34–36.9 mm; large >36.9 mm. ANOVA and Tukey post-hoc analysis revealed significantly different volar-dorsal depths (*p* < 0.05) for nearly all screws. (2) To assess biomechanical stability nine specimens were tested each; no significant differences were found between the SDLS and SLS groups.

**Conclusions:**

This feasibility study demonstrates that (1) distal radius width can be used as a predictor for distal screw length and (2) that SDLS provides mechanical stability equivalent to SLS. These results highlight the feasibility of applying SDLS screws in volar plate osteosynthesis at least in extraarticular fractures.

## Background

Volar locking plate osteosynthesis is the current standard treatment for unstable distal radius fractures (DRF) [1, 2]. Although widely applied and generally considered a safe procedure [3], complication rates of up to 18 % have been reported [4]. One of the most common complications is symptomatic extensor tendon irritation [5, 6]. Extensor tendon irritation can be caused either by direct tendon damage due to overdrilling of the dorsal cortex or by dorsal protrusion of the distal screws [7–9]. In order to reduce extensor tendon irritation, means must be found to avoid both causes.

Dorsal screw protrusion typically results from incorrect intraoperative screw length measurement. Screw length measurements are hampered by the irregular shape of the dorsal cortex and the dorsal soft tissue. A recent ultrasound study suggests that dorsal screw protrusion might occur even more often than reported. Sügün et al. [10] found 26 % of distal screws to protrude the dorsal cortex, with only 6 % becoming symptomatic. In order to prevent dorsal screw protrusion, both the AO Foundation [11] and “Campbell’s Operative Orthopaedics” [12] now recommend choosing distal screw length two to four millimeters shorter than measured. Recent experimental evidence has supported these recommendations: studies indicate that 75 % of distal screw lengths provide similar primary stability to full-length unicortical distal screws (100 %) in extraarticular distal radius fractures [13–15]. Consequently, dorsal screw protrusion can be avoided by choosing distal screws up to 25 % shorter than measured without compromising the mechanical stability.

However, no study has yet tried to overcome the problem of primary extensor tendon damage due to overdrilling. While the use of self-drilling locking screws (SDLS) would eliminate the necessity of pre-drilling, they require the ability to preoperatively determine distal screw length. Additionally, their biomechanical stability has to be comparable to standard locking screws (SLS).

When using SDLS pre-drilling is not required and hence screw length cannot be measured directly during the operation. Therefore, distal screw length has to be determined prior to surgery. The authors are not aware of any study assessing screw length preoperatively. Preoperative screw length assessment based on posterior-anterior (pa) radiographs appears possible, if the following two prerequisites are met: First, radius width (measured from pa radiographs) correlates to radius depth, i.e. distal screw length. Second, the distal screw orientation is known. Ljungquist et al. [16] have shown recently that the lunate depth can be used as a predictor. Due to its dependency on a preoperative CT image, we chose to use the distal radius width, which can be measured easily on regular radiographs. Measurements should be conducted on true pa radiographs centered on the distal radius, as outlined in a previous study [17]. This study showed a high correlation between distal radius width and depth. This indicates a possible correlation between distal pa-radius width and distal screw length, in case the screw orientation is known. The use of a drill-guide block could standardize distal screw orientation.

While there is experience with SDLS in other medical fields [18, 19] they have not yet been used in volar plate osteosynthesis for DRF. Consequently it is yet unknown, whether SDLS provide sufficient stability.

Overall, extensor tendon irritations can only be eliminated if both dorsal screw protrusion and overdrilling of the dorsal cortex are avoided. As outlined above, preoperative distal screw length determination seems possible. If SDLS prove to have similar stability to SLS, their application could be possible and would utterly eliminate both causes of extensor tendon irritation. A further advantage of this approach would be the reduction of operation time and a possible increase in overall patient satisfaction.

Therefore, we have divided this work into two parts, to test the prerequisites for the use of SDLS in DRF outlined above independently:

- Part 1: Is the distal radius width a reliable predictor for distal screw length?
- Part 2: Do SDLS provide sufficient mechanical stability for volar plate osteosynthesis of DRFs?

## Methods

The ethics committee of the University Hospital Munich, LMU, approved the study (LMU #409-13). 22 pairs of fresh-frozen radii were obtained from the Centre of Anatomy and Cell Biology, Medical University of Vienna, Austria. Following thawing, the radii were cut to 14 cm lengths. High-resolution peripheral computer tomography (HRpQCT) scans (XtremeCT, Scanco Medical AG, Switzerland) were performed and bone mineral density (BMD) and bone mineral content (BMC) were calculated [20]. Specimens were excluded if the scans revealed bone lesions, previous fractures or severe osteoarthritis.

### Part 1: Distal radius width as a predictor for screw length

**Fig. 1:**
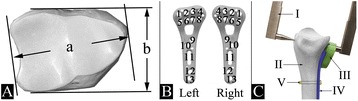
Outline of the morphometric and distal screw length measurements. **A**) Maximum width (*a*) and maximum depth (*b*); **B**) Screw numeration for right and left plate; **C**) Adapted digital caliper (*I*), distal radius (*II*), distal drill guide block (*III*), volar plate (*IV*), screw to fix plate to bone (*V*)

We evaluated whether the distal radius width can serve as a reliable predictor of distal screw length if the screw orientation is known. Maximum distal radius width and depth (Fig. 1A) were measured using a digital caliper. Distal volar polyaxial locking plates (Aptus 2.5, A-4750.61/2, Medartis Inc., Basel, Switzerland) fitted with a distal drill-guide block (Fig. 1C III, Medartis A-2723.01/02) were used for all measurements; the use of a drill-guide block is a prerequisite to ensure predictability and repeatability of the screw trajectory. Plates were placed on the bone, where they showed the best fit just proximal to the watershed line and fixed using a non-locking screw (Fig. 1C V). Plate positioning was subjective on purpose, to simulate intraoperative variance in plate positioning. Each distal screw length (Fig. 1B wholes #1–5 and 8) was then measured; a digital caliper (Fig. 1C I) with adaptations for precise positioning was used. All measurements were recorded for each specimen.

Using the above data, statistical analyses were performed to determine whether correlations exist between geometric dimensions and whether these can be clustered into size groups:

- Intra-bone linear dependency between distal radius width and depth
- Cluster analysis for distal width to identify distal radius width groups
- Descriptive screw length statistics for each screw hole within each size cluster

As a next step we evaluated whether a standard screw length could be defined per hole and within each size cluster. The evaluation was based on two aspects:

- Dorsal screw protrusion must be avoided. Therefore, the shortest screw per hole was chosen as standard length within each cluster.
- Sufficient biomechanical stability must be achieved. Based on previous studies a screw length of 75 % of the volar dorsal distance was deemed sufficient [15].

Statistics were calculated using SPSS 21.0 (IBM Company). Tests performed include the Kolmogorov-Smirnov Test, standard descriptives, Pearson correlations and a hierarchical cluster analysis using the Ward’s method and the squared Euclidean distance. Group differences were calculated using ANOVA and Tukey post hoc test (level of significance α = 0.05).

### Part 2: Mechanical stability of SDLS in DRF

In the second part of this study, we aimed to evaluate the biomechanical performance of SDLS in volar plate osteosynthesis. Therefore the primary stability of SDLS was compared to SDL using a biomechanical worst-case scenario.

**Fig. 2:**
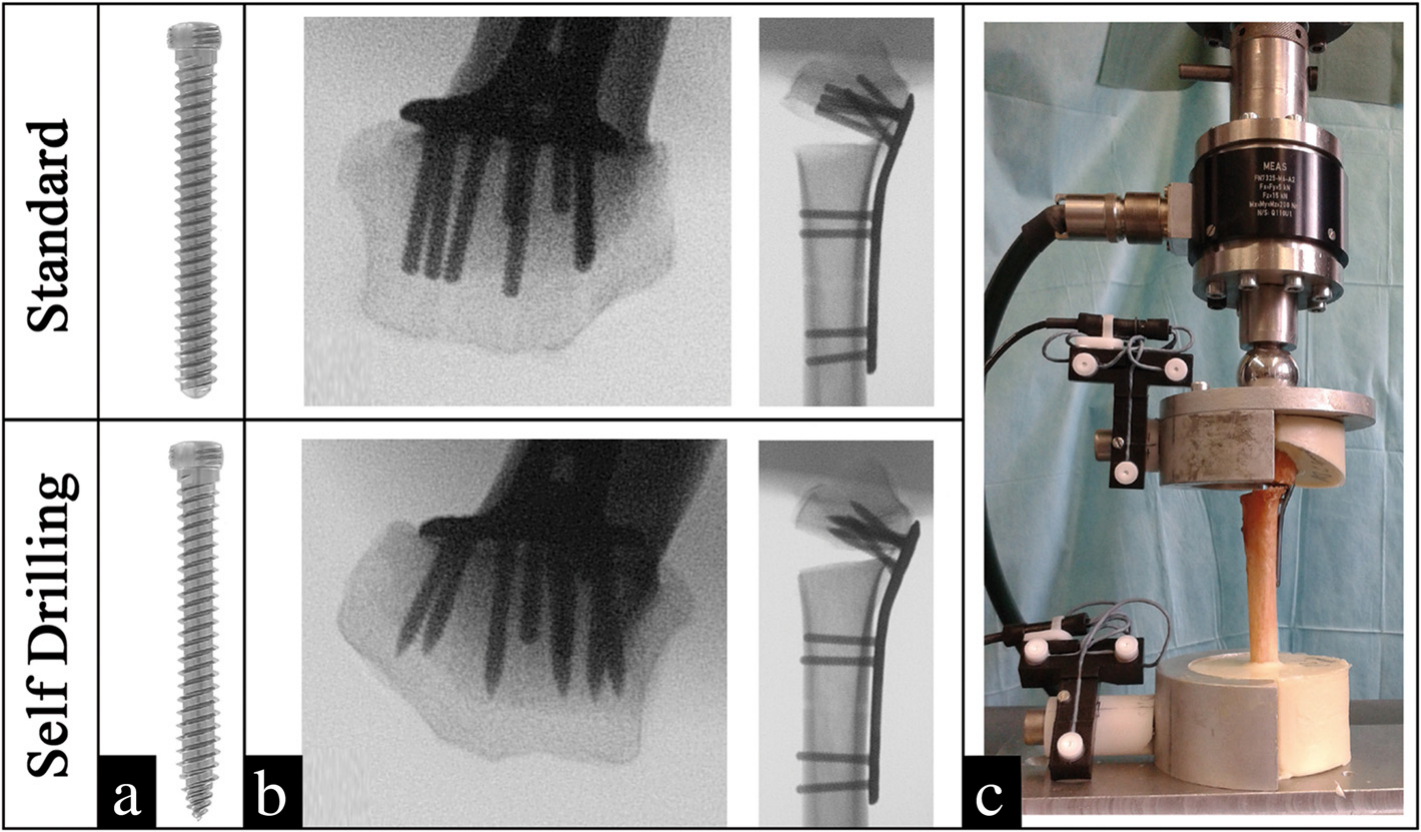
Outline of the two groups, the screws used and the final setup. **a**) Individual screws; **b**) Tangential (skyline)- and lateral views of the prepared specimens; **c**) Biomechanical test-setup (mounted with only one container shell for illustration)

For biomechanical testing eleven pairs of radii were randomly picked from the original 22. These were randomized pair-wise and side-alternating into the SLS and the SDLS group respectively. The pair-wise study design intended to ensure similar age and bone quality on both groups. 75 % distal screw length was chosen for both groups simulating a worst-case screw length scenario. Based on the initial screw length measurements, 75 % distal screws length (Fig. 1B screws #1–5, 8) were calculated and rounded to the next available screw length (2 mm increments). For both groups, distal screws were inserted using the drill-guide block. In the SLS group standard locking screws (Medartis A-5750) were inserted following pre-drilling (Fig. 2a/2b); for the SDLS group self-drilling locking screw prototypes (Fig. 2a), manufactured by Medartis, were used and inserted without pre-drilling (Fig. 2b). Plates were additionally fixed to the radius shaft with three proximal locking screws (Fig. 1B, screws # 9, 12, 13).

The herein applied, standardized, best evidence biomechanical fracture model has been descried in detail previously [21]. In short, dorsal fracture comminution was simulated by a 10 mm dorsal wedge osteotomy [22]. Specimens were aligned in a custom-made aluminum jig and 40 mm of the shaft and a shallow edge of the distal articular surface were embedded in polyurethane (SG141/PUR145, FDW Handelsgesellschaft, Austria) as outlined in Fig. 2c.

The embedded specimens were mounted to the material testing machine (Z010/TN2A, Zwick GmbH & Co. KG, Ulm, Germany). A CMS20S ultrasound motion tracking system (Zebris Medical GmbH, Isny im Allgäu, Germany) was installed to assess residual fragment tilt to quantify plastic deformation. The final setup is outlined in Fig. 2c. Axial load to failure tests were performed at a rate of 1 mm/sec until either 3 mm displacement or a 20 % drop in force; a preload of 10 N and 10 preconditioning cycles at 0.2 mm displacement were applied [21, 23].

Primary stability was assessed based on maximum force, elastic limit, stiffness, and residual distal fragment tilt. Residual tilt was computed based on the initial and final marker positions of the motion tracking system using a rigid registration according to Veldpaus et al. [24]. The remaining parameters were calculated automatically using custom Python scripts based on the load-displacement curves.

Statistics were calculated using SPSS 21.0 (IBM Company). Independent sample t-tests were conducted following Shapiro-Wilk and Levene tests to verify sample normality and variance equality. Due to multiple testing, a Bonferroni correction was applied (*p* < 0.0125). Effect size was calculated using Cohen’s d (*d* = 0.2 small effect, *d* = 0.5 medium effect, *d* = 0.8 large effect) [25].

## Results

### Part 1: Distal radius width as a predictor for screw length

38 radii with a mean age of 79 ± 12 years (42 % female) were included in the final analysis. Three pair of radii had to be excluded due to previous fractures. The prerequisite of normality was met. Distal radius width and depth were highly correlated (*R*^*2*^ = 0.79; *p* < 0.001); standard descriptive statistics for distal width, depth and distal screw length are presented in Table 1.

**Table 1:** Standard descriptive statistics for distal radius width, depth and distal screw length [mm]

	Depth	Width	Screw #1	Screw #2	Screw #3	Screw #4	Screw #5	Screw #8
Mean ± SD	25.4 ± 2.5	35.2 ± 2.8	20.6 ± 2.4	24.1 ± 1.8	24.0 ± 2.3	23.3 ± 1.8	21.7 ± 2.1	23.1 ± 2.1
95 % CI	24.5–26.2	34.3–36.1	19.8–21.4	23.5–24.7	23.2–24.7	22.7–23.9	21.0–22.4	22.4–23.8
Min	22.0	30.0	15.0	21.0	20.0	20.5	18.5	18.0
Max	30.0	40.0	26.0	28.0	28.0	27.0	26.0	27.0

*SD*, Standard deviation, *95 % CI* 95 % confidence interval, *Min* Minimum, *Max* Maximum

Using cluster analysis three homogeneous clusters for distal radius width were defined:

$$ \mathrm{Small}: < 34\ \mathrm{mm};\ \mathrm{Medium}:\ 34\ \hbox{--}\ 36.9\ \mathrm{mm};\ \mathrm{Large}: > 36.9\ \mathrm{mm}. $$

**Fig. 3:**
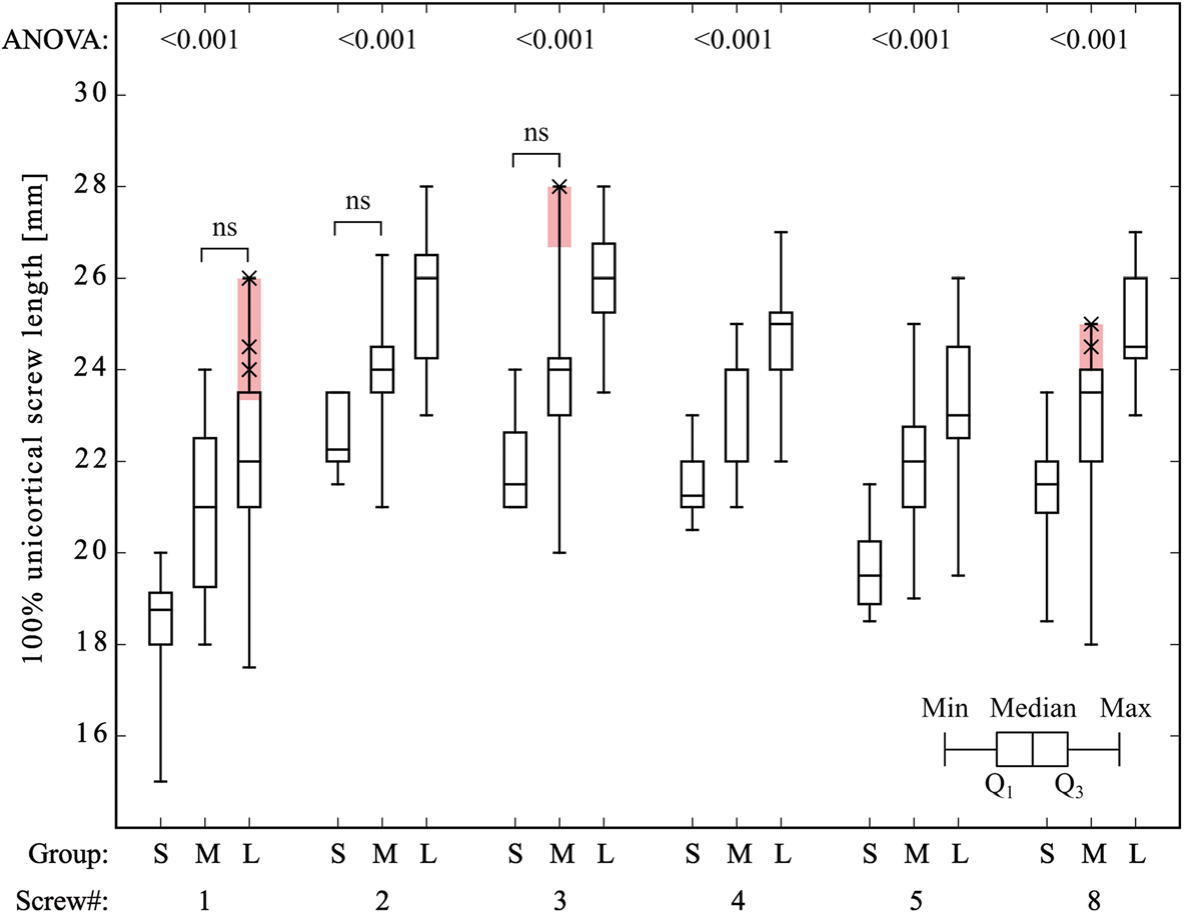
Box-plots for each screw length in each group. S: Group small; M: Group medium; L: Group large; Red box and crosses: Screws falling short of the “safe screw length corridor”; ANOVA: Overall significant differences between each group for each screw. Tukey post hoc test revealed significant differences for all groups, but those highlighted as “ns” (= non significant)

In a next step box-plots were generated for every screw position in all clusters to define standard screw lengths (Fig. 3). The shortest screw for every screw hole and cluster was used as the standard screw length; using this methodology out of 228 screws only 7 fell short of the “safe screw length corridor” (Fig. 3 red boxes); in these cases the screw length chosen was less than 75 % of the actual length measured.

### Part 2: Mechanical stability of SDLS in DRF

Of the eleven pairs of radii selected for biomechanical testing two pairs had to be excluded due to previous fractures. The remaining 9 pairs with a mean age of 71 ± 8 years (33 % female) were tested successfully. Normality and variance equality criteria were met. BMD (237 ± 72 vs. 233 ± 76 mgHA/cm^3^; *p* = 0.6) and BMC (1295 ± 457 vs. 1315 ± 533 mgHA; *p* = 0.6) did not differ significantly between the two groups. Table 2 provides group-wise statistics for all biomechanical outcome parameters. Overall the SDLS group showed better mechanical properties but differences were not statistically significant. The effect size calculation indicated a medium effect for maximum force (*d* = 0.58) [26].

**Table 2:** Group statistics for the biomechanical outcome parameters

Source	Parameter	Group	Mean	SD
Load-displacement-Curves	Stiffness [N/mm]	SLS	833	160
		SDLS	904	279
	Elastic Limit [N]	SLS	205	42
		SDLS	259	106
	Max Force [N]	SLS	632	273
		SDLS	768	188
Motion Tracking	Residual Tilt [°]	SLS	5.7	1.3
		SDLS	5.6	0.9

No significant differences were found for any parameter

*Max Force* maximum force, *N* Newton, *mm* millimeter, ° degree, *SD* standard deviation, *ns* not significant

## Discussion

For the first time, this study demonstrated the feasibility of SDLS volar plate osteosynthesis for DRF. Both prerequisites, i.e. screw length estimation based on distal radius width and primary stability of SDLS were fulfilled. First, three distal radius width clusters were identified and the definition of a standard screw length for each screw hole in each group appeared possible. Second, SDLS provided equal primary stability in volar locking plate osteosynthesis compared to SLS.

### Part 1: Evaluation of preoperative screw length determination based on distal radius width

Based on the data presented, the definition of group- and screw hole specific standard screw lengths appears feasible. When applying the “safe screw length corridor” concept, only seven out of 228 distal screws would have fallen short of the 75 % lower bound (Fig. 3 red boxes): in five specimens one screw each and in one sample two screws were shorter than 75 % of the volar-dorsal distance. Based on the results by Wall et al., it is questionable whether this would have a measurable impact on the overall stability.

We are aware that our cluster- and screw length analysis is based on a rather small sample size. And while the observed mean radius width is similar to values reported in literature [17, 27, 28], the sample’s width range (30 mm–40 mm) is not. Previous studies published width ranges of 24 mm to 46 mm [17]. The small width range is most likely due to the limited sample size and the use of paired radii, which further reduces the morphometric variety. Additionally, morphologic population differences may also influence the results [17, 27, 29]. To predict cluster- and screw hole specific screw lengths, this study should be repeated in larger samples to observe differences associated in gender and ethnicity. Also, distal screw length depends on the plate position. While most manufacturers recommend placing the plate just proximal to the watershed-line, fracture pattern and the plate’s shape influence the intraoperative plate position. We tried to account for this variability at least partially by not standardizing the plate position. Finally, future studies will have to assess the suitability of these pa-width measurements in the fractured case. Although fracture dislocation occurs primarily dorsally in extra-articular fractures (AO-23-A3), which should only has a limited affect on the pa-distal radius width measurement, control measurements should be taken in case the fracture is reduced prior to surgical treatment.

Despite the limitations stated above, the data presented suggest that defining standard screw lengths based on the distal radius width is feasible. While the use of screws with standardized, predetermined lengths should mostly eliminate dorsal screw protrusions, a final control using the radiographic skyline view [30, 31] or a similar technique would remain a prerequisite.

### Part 2: Primary stability of SLS compared to SDLS in a mechanical worst-case scenario

SDLS in volar locking plate osteosynthesis for DRF were compared to SLS in a worst-case biomechanical test setup. Using axial load to failure tests [21, 22] we were able to show a similar primary stability for SDLS compared to SLS.

The stiffness and maximum forces observed were in agreement with values previously published, ranging from 450 N/mm to 800 N/mm and 300 N to 1050 N, respectively [21, 32, 33]. All samples tested easily withstood the critical load of 250 N occurring during early rehabilitation [34, 35]. The mean stiffness, elastic limit and maximum forces were higher in the SDLS group compared to the SLS group. While the differences were not statistically significant, this might indicate a higher primary stability using the SDLS. This is in line with previous single screw pull-out tests reporting superior performance of SDLS compared to SLS [18, 19, 36].

Several limitations remain. The use of isolated radii (without ulna and the surrounding soft tissue) may oversimplify the biomechanical situation; however, it is the method typically employed in biomechanical testing and eliminates confounding factors [21, 32, 33]. A simple uniaxial loading in compression was used; while this will still cause shear loads and moments, the varying in-vivo loading patterns due to different hand postures will not be fully represented [37]. Finally, only one extra-articular fracture type (AO-23-A3) was tested. Whether similar results can be obtained for intra-articular fractures needs to be investigated, but we assume that more complex fracture patterns require the use of individually oriented screws. This would prevent the use of a drill guide block (which sets a predefined axis for every screw) and thus the use of screws with predetermined lengths.

## Conclusion

Out tests show that SDLS provide sufficient primary mechanical stability in volar locking plate osteosynthesis. Additionally, distal radius width appears to be a valid predictor for screw hole specific standard distal screw length. While a larger sample size might be necessary to refine the distal radius width groups, the results obtained from this limited set give a first orientation. The results of this study highlight the feasibility of applying SDLS in volar plate osteosynthesis for the distal radius at least for extraarticular fractures saving not only operation time, but also eliminating the causes of extensor tendon irritations.

## Abbreviations

ANOVA: Analysis of variance
AO-23 A3: AO-Classification: Distal radius fracture, dorsally displaced, multifragmentary
BMC: Bone mineral content
BMD: Bone mineral density
CT: Computer Tomography
DRF: Distal radius fracture
HRpQCT: High-resolution peripheral computer tomography
SDLS: Self-drilling locking screws
SLS: Standard locking screws
